# Age Modulates Fe_3_O_4_ Nanoparticles Liver Toxicity: Dose-Dependent Decrease in Mitochondrial Respiratory Chain Complexes Activities and Coupling in Middle-Aged as Compared to Young Rats

**DOI:** 10.1155/2014/474081

**Published:** 2014-05-06

**Authors:** Yosra Baratli, Anne-Laure Charles, Valérie Wolff, Lotfi Ben Tahar, Leila Smiri, Jamal Bouitbir, Joffrey Zoll, Mohsen Sakly, Cyril Auger, Thomas Vogel, Hafedh Abdelmelek, Olfa Tebourbi, Bernard Geny

**Affiliations:** ^1^EA 3072, Mitochondries, Stress Oxydant et Protection Musculaire, Fédération de Médecine Translationnelle de Strasbourg, Université de Strasbourg, 67000 Strasbourg, France; ^2^Laboratoire de Physiologie Intégrée, Faculté des Sciences de Bizerte, Université de Carthage, 7021 Jarzouna, Tunisia; ^3^Laboratoire de Synthèse et Structures de Nanomatériaux, UR11ES30, Faculté des Sciences de Bizerte, Université de Carthage, 7021 Jarzouna, Tunisia; ^4^Service de Physiologie et d'Explorations Fonctionnelles, Pôle de Pathologie Thoracique, NHC, 67000 Strasbourg, France; ^5^UMR CNRS 7213-Laboratoire de Biophotonique et Pharmacologie, Faculté de Pharmacie, Université de Strasbourg, 74 route du Rhin, 67401 Illkirch, France

## Abstract

We examined the effects of iron oxide nanoparticles (IONPs) on mitochondrial respiratory chain complexes activities and mitochondrial coupling in young (3 months) and middle-aged (18 months) rat liver, organ largely involved in body iron detoxification. Isolated liver mitochondria were extracted using differential centrifugations. Maximal oxidative capacities (*V*_max_, complexes I, III, and IV activities), *V*_succ_ (complexes II, III, and IV activities), and *V*_tmpd_, (complex IV activity), together with mitochondrial coupling (*V*_max_/*V*_0_) were determined in controls conditions and after exposure to 250, 300, and 350 **μ**g/ml Fe_3_O_4_ in young and middle-aged rats. In young liver mitochondria, exposure to IONPs did not alter mitochondrial function. In contrast, IONPs dose-dependently impaired all complexes of the mitochondrial respiratory chain in middle-aged rat liver: *V*_max_ (from 30 ± 1.6 to 17.9 ± 1.5; *P* < 0.001), *V*_succ_ (from 33.9 ± 1.7 to 24.3 ± 1.0; *P* < 0.01), *V*_tmpd_ (from 43.0 ± 1.6 to 26.3 ± 2.2 *µ*mol O_2_/min/g protein; *P* < 0.001) using Fe_3_O_4_ 350 *µ*g/ml. Mitochondrial coupling also decreased. Interestingly, 350 **μ**g/ml Fe_3_O_4_ in the form of Fe^3+^ solution did not impair liver mitochondrial function in middle-aged rats. Thus, IONPs showed a specific toxicity in middle-aged rats suggesting caution when using it in old age.

## 1. Introduction

Because of their unique properties, some nanoparticles have been approved for clinical use like iron oxide nanoparticles (IONPs). IONPs hold immense potential in a vast variety of biomedical applications such as magnetic resonance imaging (MRI), targeted delivery of drugs or genes, tissue engineering, targeted destruction of tumor tissue through hyperthermia, magnetic transfections, iron detection, chelation therapy, and tissue engineering [1–4]. Reports demonstrate that IONPs have the ability to assess focal hepatic lesions [5] and to label human hepatocytes [6].

However, nanoparticles pose a high health risk because of their ability to reach every part of the organs and tissues and their interaction with cellular functions.

Concerning NP clearance, it is known that they are primarily phagocytozed by macrophages in the liver (Kupffer cells) [7]. Thus, the largest detoxification organ of human beings, the liver, is reached by the highest amount of nanoparticles, over all the other tissues [8, 9]. Furthermore, mitochondria are considered a major cell compartment relevant to possible nanoparticle toxicity [10]. Impairment of mitochondria might be a key problem since mitochondrial dysfunction may result in reduced cellular ATP delivery, increased reactive oxygen species production, and triggering of apoptosis pathways. Accordingly, mitochondrial dysfunctions occur early in many acute or chronic diseases such as peripheral arterial or pulmonary diseases [11, 12].

Mitochondrial involvement in IONPs toxicity remains controversial and either no deleterious effects [13, 14] or mitochondrial impairments have been observed [15–17].

Concerning Fe_3_O_4_ nanoparticles, we recently reported a lack of IONPs toxicity in liver mitochondria in young rats [18]. Nevertheless, age-related accumulation of iron increases the potential for free redox-active iron, which can promote oxidative stress and mitochondrial damage [19]. More recently, [20] reported that age-dependent accumulation of mitochondrial iron may increase mitochondrial dysfunction and oxidative damage, thereby enhancing the susceptibility to apoptosis. Therefore, age might modify the susceptibility of mitochondria to iron nanoparticles (NPs).

Since, to date, no study investigated the potential effects of iron oxide nanoparticles on middle-aged mitochondria and since the liver is a key organ in iron and NPs detoxification, we investigated and compared for the first time the effects of three different concentrations of Fe_3_O_4_ nanoparticles (250, 300, and 350 *μ*g/mL) on young and middle-aged liver mitochondrial respiratory chain complexes activities and on mitochondrial coupling of phosphorylation to oxidation.

## 2. Materials and Methods

### 2.1. Materials and Reagents

Iron oxide nanoparticles were acquired from Unit of Research UR11ES30, Faculty of science of Bizerte, Tunisia. They were prepared by the polyol process starting from iron (II) acetate as metal precursor and diethylene glycol (DEG) as solvent purchased from ACROS Organics. All chemicals were used as received without further purification. Deionized water was used in these preparations. For the synthesis of the magnetite (Fe_3_O_4_) nanoparticles, an appropriate amount of iron (II) acetate precursor was added to a given volume (125 mL) of DEG to reach nominal iron cations concentration of 0.2 M. The mixture was then refluxed at a rate of +6°C min^−1^under mechanical stirring up to boiling point and then maintained at this temperature for about 2 h. The powders were washed several times with ethanol and then with acetone under ultrasonication with intermittent centrifugation and then dried in air at 50°C [18].

### 2.2. Nanoparticle Characterization Using Transmission Electron Microscopy (TEM)

The size and shape of prepared particles were analyzed on a JEOL-100-CX II transmission electron microscope (TEM) operating at 100 kV equipped with an energy dispersive spectrometer (EDX) [21].

**Figure 1:**
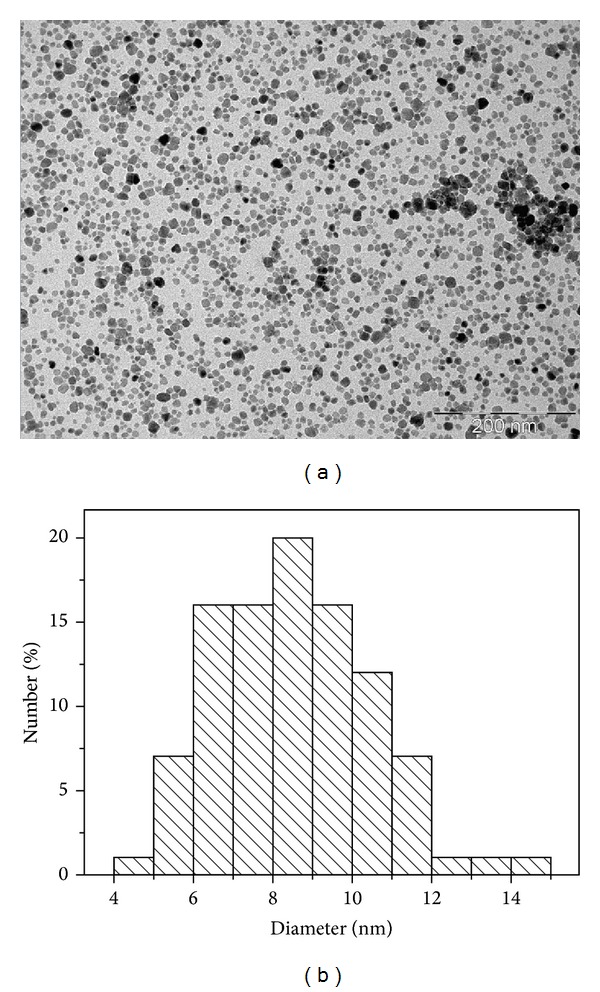
TEM image (a) and size histogram (b) of the Fe_3_O_4_ nanoparticles.

TEM imaging of Fe_3_O_4_ nanoparticles showed that the powders are constituted by roughly spherical almost nonagglomerated particles. In addition, about 250 particles have been counted for the average particle size and histogram determinations. The calculated average diameter was 9 nm with a SD of 1.6 nm (Figure 1).

### 2.3. Animals

Young (*n* = 6, age 3 months) and middle-aged (*n* = 6, age 18 months) Wistar male rats were housed in a thermoneutral environment (22 ± 2°C), on a 12 : 12 h photoperiod, and were provided food and water* ad libitum*. This investigation was carried out in accordance with the Guide for the Care and Use of Laboratory published by the US National Institute of Health and approved by the institutional animal care committee (NIH publication number 85-23, revised 1996).

Rats were submitted to general anesthesia with 3% isoflurane and oxygen (1 L/min) in an induction chamber (Minerve, Esternay, France). Anesthesia was maintained with 1.5% isoflurane and 1 L/min oxygen at under spontaneous ventilation. A midline laparotomy was performed and the liver was excised, cleaned, and then immediately used for the study of respiratory parameters.

Similarly, three additional Wistar rats aged 18 months were studied, in order to investigate the potential effect of iron oxide* per se*.

### 2.4. Extraction of Mitochondria

All operations were carried on ice. A piece of tissue was placed into buffer A containing 50 mM tris, 1 mM EGTA, 70 mM sucrose, 210 mM mannitol, and pH 7.40 at +4°C. Tissue was finely minced with scissors, placed in buffer A, and homogenized with a Potter-Elvehjem. Then, the homogenate was centrifuged at 1300 ×g for 3 min and 4°C. The supernatant was centrifuged at 10,000 ×g for 10 min and 4°C to sediment mitochondria. Finally, the mitochondrial pellet was washed twice and then suspended in 50 mM tris, 70 mM sucrose, 210 mM mannitol, and pH 7.4 at +4°C. Protein content was routinely quantified with a Bradford assay using bovine serum albumin as a standard [22]. Mitochondria were kept on ice and used within 4 h.

### 2.5. Exposure of Mitochondria to Nanoparticles or to Iron Oxide

The iron oxide nanoparticles were mixed with a solution of NaCl 9%. The mixture was then stirred vigorously and sonicated for 60 min to break up aggregates. Particle suspensions were vortexed immediately before each use. Before measurement, 3 mL of solution M containing 100 mM KCL, 50 mM mops, 1 mM EGTA, 5 mM Kpi, and 1 mg/mL bovine serum albumin (BSA) was added to the oxygraph chambers for 10 min. Then, 0.50 mg of mitochondrial protein was placed in the oxygraph chambers with 10 mM glutamate and 2.50 mM malate as substrates. The temperature was maintained at +25°C. Isolated liver mitochondria were incubated with different concentrations of Fe_3_O_4 _(0, 250, 300, and 350 *μ*g/mL) during 30 min at +25°C.

To discriminate if the results obtained might be due to the size or to a general response pattern in front of iron oxide exposure, we submitted middle-aged liver mitochondria to a Fe^3+^ solution with concentration of 350 *μ*g/mL Fe_3_O_4_. To obtain free Fe^3+^ ions a precise mass of Fe_3_O_4_ nanoparticles was dissolved in a small volume of concentrated HCl under gentle heating [23]. Two experiments were conducted* per* animal, and thus number of data was six.

### 2.6. Measurement of the Mitochondrial Respiratory Chain Complexes Activities and Mitochondrial Coupling

Maximal oxidative capacity (*V*_max⁡_) was measured by adding adenosine diphosphate (ADP). When *V*_max⁡_ was recorded, electron flow went through complexes I, III, and IV. Then complex I was blocked with amytal (0.02 mM) and complex II was stimulated with succinate (25 mM). Mitochondrial respiration in these conditions allowed determining complexes II, III, and IV activities (*V*_succ_). After that, N, N, N′, N′-tetramethyl-p-phenylenediaminedihydrochloride (TMPD, 0.50 mM) and ascorbate (0.50 mM) were added as artificial electron donors to cytochrome c. In these conditions, the activity of cytochrome c oxidase (complex IV) was determined as an isolated step of the respiratory chain (*V*_tmpd_) [24].

Mitochondrial coupling (coupling of phosphorylation to oxidation) was determined by calculating the acceptor control ratio (ACR), ratio between ADP-stimulated respiration (*V*_max⁡_) over basal respiration (without ADP) with glutamate and malate as substrate (*V*_0_), as previously reported by Mansour et al. 2012 [25].

### 2.7. Statistical Analysis

Results are expressed as mean ± SEM. Statistical analyses were performed using one-way ANOVA followed by a Tukey posttest. The unpaired *t*-test was used to compare young and old rats. Statistical significance required a *P* < 0.05. Statistical analyses were performed using GraphPad Prism 5 (GraphPad Software, Inc., San Diego, CA, USA).

## 3. Results

### 3.1. Effects of Age on Mitochondrial Respiratory Chain Function and on Mitochondrial Coupling

As shown in Table 1, there was no difference in mitochondrial oxygen consumptions between young (3 months) and middle-aged (18 months) control rats liver. Thus, the maximal oxidative capacity was similar in young versus middle-aged rats (29.8 ± 5.6 and 30.0 ± 1.6 *μ*mol O_2_/min/g protein, respectively). This holded true for *V*_succ_ (young: 35.0 ± 3.7 versus 33.9 ± 1.7 *μ*mol O_2_/min/g protein) and for *V*_tmpd_ (young: 50.0 ± 6.2 versus 43.0 ± 1.6 *μ*mol O_2_/min/g protein).

Finally, mitochondrial coupling was similar in both young and middle-aged rats (9.7 ± 2.6 versus 10.5 ± 1.3; *P* = NS).

### 3.2. Effects of IONPs on Young Liver Mitochondrial Respiratory Chain Complexes Activities

**Figure 2:**
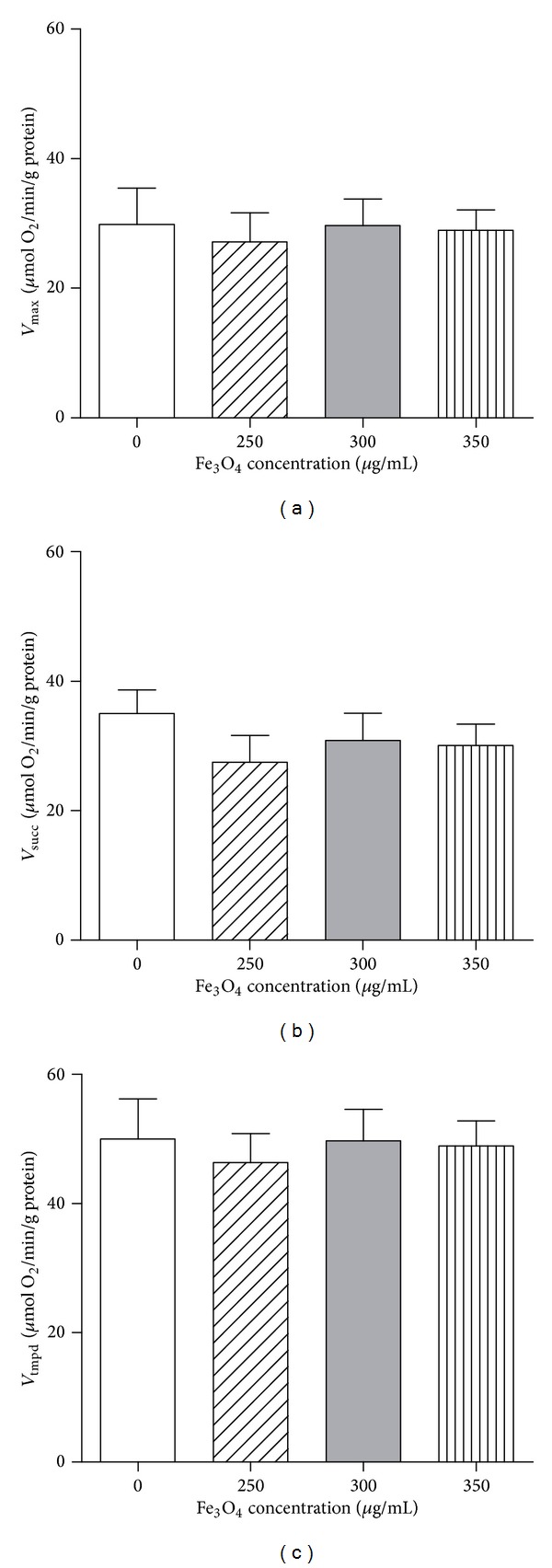
Effects of iron oxide nanoparticles (Fe_3_O_4_) on young liver mitochondrial respiratory chain complexes activities. (a) *V*_max⁡_ reflects complexes I, III, and IV activities and is measured using glutamate and malate. (b) *V*_succ_ reflects complexes II, III, and IV activities and is measured using succinate. (c) *V*_tmpd_ reflects complex IV activity and is measured using TMPD and ascorbate as mitochondrial substrates. Data are means ± SEM

*Complexes I, III, and IV Activities.* The *V*_max⁡_ in the group treated with increasing doses of IONPs (250, 300, and 350 *μ*g/mL) was not modified as compared to the control group (27.2 ± 4.5; 29.7 ± 4.1; 29.0 ± 3.1 versus 29.8 ± 5.6 *μ*mol O_2_/min/g protein) (Figure 2(a)).

*Complexes II, III, and IV Activities. V*_succ_ was unchanged whatever the dose of IONPs (27.5 ± 4.2, 30.9 ± 4.2, and 30.1 ± 3.3 for Fe_3_O_4 _ 250, 300, and 350, respectively, as compared to control values 35.0 ± 3.7 *μ*mol O_2_/min/g protein) (Figure 2(b)).

*Complex IV Activity. V*_tmpd_, reflecting complex IV activity, was not modified after IONPs treatment as compared with control group (46.3 ± 4.5, 49.7 ± 4.9, and 50.0 ± 3.8 for Fe_3_O_4 _ 250, 300, and 350, respectively, versus 50.0 ± 6.2 *μ*mol O_2_/min/g protein) (Figure 2(c)).

Taken together, these data support that Fe_3_O_4_ nanoparticles, whatever the doses used, failed to alter any of the liver mitochondrial respiratory chain complexes activities in young rats.

### 3.3. Effects of IONPs on Middle-Aged Liver Mitochondrial Respiratory Chain Complexes Activities

When the middle-aged liver rats were exposed to different concentrations of IONPs, the *V*_max⁡_, *V*_succ_, and *V*_tmpd_ decreased significantly. 

*Complexes I, III, and IV Activities.* The maximal oxidative capacities, *V*_max⁡_, reflecting I, III, and IV activities significantly decreased whatever the dose of IONPs. At 250 *μ*g/mL, *V*_max⁡_ was decreased as compared to the control group (24.4 ± 1.4 versus 30 ± 1.6 *μ*mol O_2_/min/g protein; *P* < 0.05). *V*_max⁡_ at 300 and 350 *μ*g/mL were also decreased as compared to control values (23.8 ± 1.0 and 17.9 ± 1.5 for Fe_3_O_4_ 300 and Fe_3_O_4_ 350 *μ*g/mL, respectively, versus CON: 30 ± 1.6 *μ*mol O_2_/min/g protein; *P* < 0.001).

**Figure 3:**
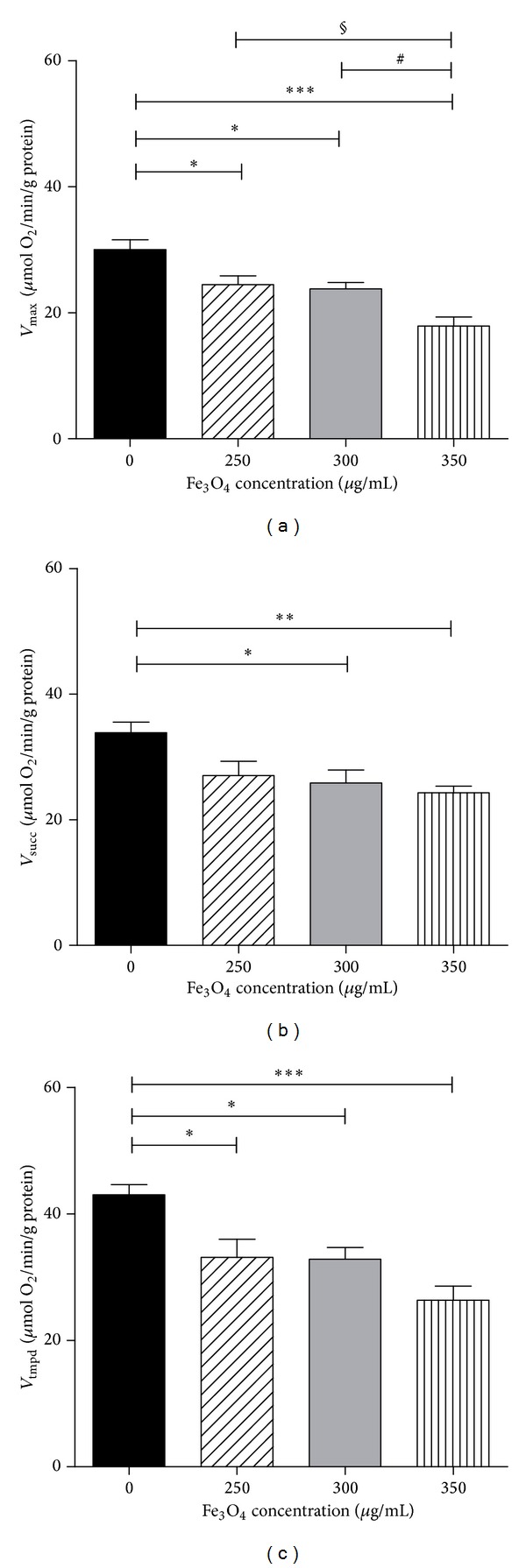
Effects of iron oxide nanoparticles (Fe_3_O_4_) on middle-aged liver mitochondrial respiratory chain complexes activities. (a) *V*_max⁡_ reflects complexes I, III, and IV activities and is measured using glutamate and malate. (b) *V*_succ_ reflects complexes II, III, and IV activities and is measured using succinate. (c) *V*_tmpd_ reflects complex IV activity and is measured using TMPD and ascorbate as mitochondrial substrates. Data are means ± SEM (one-way ANOVA followed by Tukey). *P < 0.05; **P < 0.01; ***P < 0.001 compared to control. ^#^ *P* < 0.05 350 *μ*g/mL compared to 300 *μ*g/mL. ^§^ *P* < 0.05 350 *μ*g/mL compared to 250 *μ*g/mL.

The *V*_max⁡_ decrease was greater when using higher IONPs concentration. Thus *V*_max⁡_ was significantly lower after Fe_3_O_4_ 350 *μ*g/mL as compared to 300 and 250 *μ*g/mL (Figure 3(a)).

*Complexes II, III, and IV Activities.*  At 250 *μ*g/mL, *V*_succ_, reflecting complexes II, III, and IV activities, tended to decrease as compared to the control group (27.0 ± 2.3 versus 33.9 ± 1.7 *μ*mol O_2_/min/g protein). The statistical significance was reached at 300 *μ*g/mL and 350 *μ*g/mL (Fe_3_O_4_ 300 *μ*g/mL: 25.8 ± 1.0 versus CON: 33.9 ± 1.7 *μ*mol O_2_/min/g protein; *P* < 0.05) and Fe_3_O_4_ 350 *μ*g/mL (24.3 ± 1.0 versus 33.9 ± 1.7 *μ*mol O_2_/min/g protein; *P* < 0.01) (Figure 3(b)). 

*Complex IV Activity. V*_tmpd_, reflecting complex IV activity, decreased significantly after exposure to Fe_3_O_4_ 250 *μ*g/mL as compared to the control group (33.0 ± 2.9  versus 43.0 ± 1.6 *μ*mol O_2_/min/g protein; *P* < 0.05). Similarly, *V*_tmpd_, decreased when exposed to 300 *μ*g/mL of Fe_3_O_4_ (32.9 ± 1.9  versus 43.0 ± 1.6 *μ*mol O_2_/min/g protein; *P* < 0.05) and to 350 *μ*g/mL (26.3 ± 2.2 versus 43.0 ± 1.6 *μ*mol O_2_/min/g protein; *P* < 0.001) (Figure 3(c)).

### 3.4. Effects of IONPs on Liver Mitochondrial Coupling in Young and Middle-Aged Rats

In young liver, the acceptor control ratio (*V*_max⁡_/*V*_0_), representing the degree of coupling between oxidation and phosphorylation, was not changed after IONPs treatment (8.4 ± 1.8, 9.1 ± 1.7, and 8.0 ± 1.2 after, respectively, Fe_3_O_4 _ 250, 300, and 350 *μ*g/mL, as compared to control 9.7 ± 2.6).

**Figure 4:**
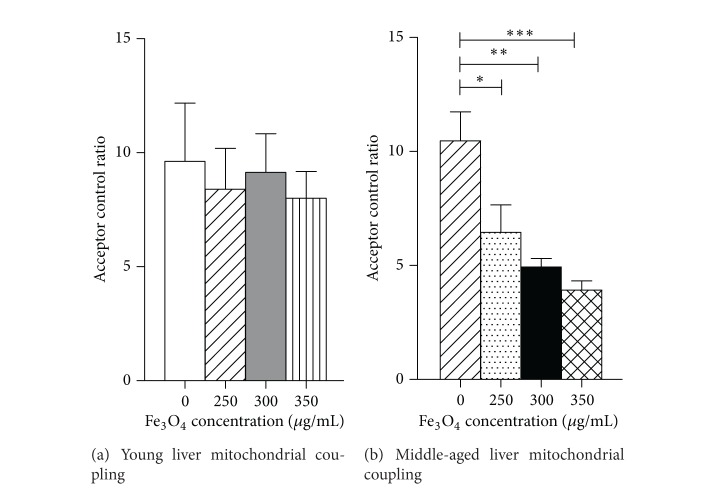
Effects of iron oxide nanoparticles (Fe_3_O_4_) on (a) young and (b) middle-aged liver mitochondrial coupling. Data are means ± SEM (one-way ANOVA followed by Tukey). *P < 0.05; **P < 0.01; ***P < 0.001 compared to control.

On the other hand, interestingly, IONPs decreased significantly mitochondrial coupling in middle-aged liver, as compared to controls. Thus, the ACR was lower when mitochondria were exposed to 250 *μ*g/mL Fe_3_O_4, _as compared to control (6.4 ± 1.2 versus 10.5 ± 1.3; *P* < 0.05). Moreover, ACR was also decreased at 300 *μ*g/mL (5.0 ± 0.4 versus 10.5 ± 1.3; *P* < 0.01) and at 350 *μ*g/mL (Fe_3_O_4_ 350 *μ*g/mL: 3.9 ± 0.4 versus CON: 10.5 ± 1.3; *P* < 0.001) (Figure 4).

### 3.5. Effects of Iron Oxide Not in Its Particulate form on Middle-Aged Liver Mitochondrial Respiratory Chain Complexes Activities and Coupling

**Figure 5:**
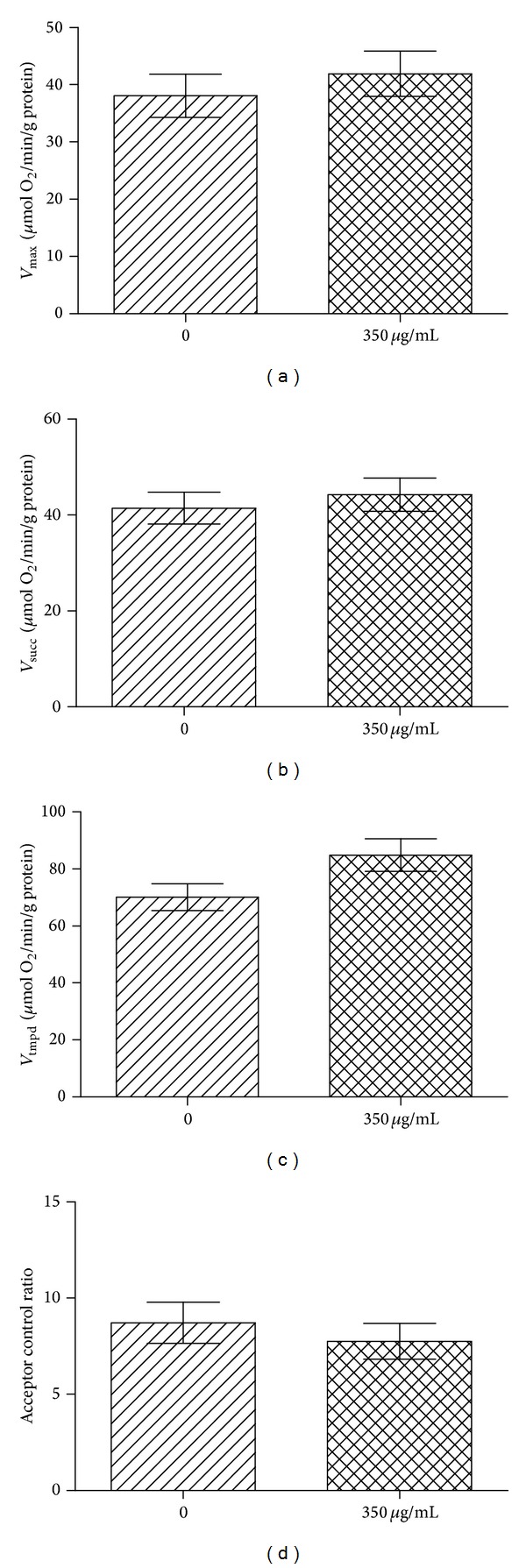
Effects of 350 *μ*g/mL of Fe_3_O_4 _but not in its particulate form on middle-aged liver mitochondrial respiration and coupling. (a) *V*_max⁡_ reflects complexes I, III, and IV activities and is measured using glutamate and malate. (b) *V*_succ_ reflects complexes II, III, and IV activities and is measured using succinate. (c) *V*_tmpd_ reflects complex IV activity and is measured using N, N, N′, N′-tetramethyl-p-phenylenediaminedihydrochloride (TMPD) and ascorbate as mitochondrial substrates. (d) Acceptor control ratio reflects the mitochondrial coupling. Data are means ± SEM.

When the middle-aged liver rats were exposed to 350 *μ*g/mL Fe_3_O_4_, *V*_max⁡_ (41.90 ± 3.93 versus 38.08 ± 3.77 *μ*mol O_2_/min/g protein), *V*_succ_ (44.23 ± 3.443 versus 41.42 ± 3.303 *μ*mol O_2_/min/g protein), *V*_tmpd_ (84.90 ± 5.75 versus 70.08 ± 4.71 *μ*mol O_2_/min/g protein), and ACR (7.70 ± 1.15 versus 8.72 ± 1.06) were not significantly modified (Figure 5).

## 4. Discussion

The main results of this study are to demonstrate that, unlike young animals, middle-aged rats are sensitive to iron oxide nanoparticles (IONPs). Indeed, liver mitochondrial respiration chain complexes I, II, III, and IV activities are altered whatever the concentration of Fe_3_O_4 _used. Further, 250, 300, and 350 *μ*g/mL of Fe_3_O_4 _specifically impair liver mitochondrial coupling in middle-aged rats. Such impairment is not observed when using 350 *μ*g/mL of Fe_3_O_4 _but not in its particulate form.

*Young and Middle-Aged Rats Present with Similar Baseline Mitochondrial Respiratory Chain Complexes Activities in the Liver.* Several studies indicate that mitochondria are one of the major sources of reactive oxygen species (ROS) and, in turn, are the most adversely affected organelles during aging [26]. In fact, mitochondria from aged tissue use oxygen inefficiently, which impairs ATP synthesis and results in increased oxidant production [27]. Oxidative damage mainly concerns the activities of electron transport complexes of the inner mitochondrial membrane [28], which are specifically modified during aging [29, 30].

Accordingly, in rat liver, previous reports have found aging-related decrease in electron transfer activity in complex I or complexes I and IV in young (4 months) versus old (30 months) rats [31–34]. On the other hand, some studies found no change in the respiratory chain activity with age. Thus, Bakala et al. [35] observed no significant difference in the respiratory chain activity with age between 10-month-old and 27-month-old rats, whatever the substrate used.

This is consistent with our results and might be explained by differences in age range or animals being only middle-aged.

*Iron Oxide Nanoparticles Impair Liver Mitochondrial Respiratory Chain Complexes Activities in Middle-Aged but Not in Young Rats.* Concerning young rats, iron oxide nanoparticles failed to impair any of the liver mitochondrial respiratory chain complexes activities. Thus, mitochondrial oxygen consumption in young liver was not altered by IONPs and, similarly, mitochondrial coupling remained in the normal range after Fe_3_O_4_ exposure. This is in agreement with our previous findings [18].

To the best of our knowledge, no data are available concerning potential related effects of high levels of iron oxide nanoparticles and age on mitochondria. This is particularly interesting since the liver is a major iron storage organ [36, 37]. Very interestingly, the data were different when middle-aged liver rats were exposed to the same IONPs concentrations than the young ones. In middle-aged liver rats, IONPs at 250, 300, and 350 *μ*g/mL significantly decreased *V*_max⁡_, *V*_succ_, and *V*_tmpd_ corresponding together to complexes I, II, III, and IV activities of the mitochondrial respiratory chain. IONPs exposure also decreased mitochondrial coupling.

Several mechanisms might explain these results like increased fragility of older mitochondria and iron accumulation.

First, the fragility of mitochondria seems to increase in function of age. Thus, studies reported that mitochondria isolated from the organs of aged animals are also aged in terms of cytosolic and mitochondrial oxidative stress and losses of enzymatic activities [32]. Accordingly, studies demonstrated that aging induces the loss of mitochondrial function in liver of rodents and monkeys [38–40].

Similarly studies investigated the effects of iron accumulation on mitochondrial integrity and function with age [20, 41, 42]. Investigating the pharmacokinetics of IONPs in rats, Schnorr et al. [43] demonstrated that the half-life and the resulting signal changes in blood and liver vary significantly with age. Thus, iron accumulation which is considered a feature of the aging process [44–46] might be associated with a mitochondrial iron increase. In particular under conditions of cellular stress, this may be a potential causative factor of age-related mitochondrial dysfunction [20, 42, 47, 48]. Mitochondrial damage by excessive cellular iron appears as an intrinsic factor contributing to the permeability pore transition and bioenergetics function decline of mitochondria, which can potentially lead to cellular senescence and tissue degeneration [49–51].

Particularly, in accordance with our data of a NPs-related impairment of complex IV, [52] demonstrated that the interaction of bare Fe_3_O_4_ NPs with cytochrome c leads to the reduction of the protein. Moreover, [53] using human hepatoma cells showed a dose-dependent decrease in mitochondrial membrane potential after exposure to Fe_3_O_4_ NPs and at high concentration (1 mg/mL) the cytochrome c protein expression decreased dramatically. Accordingly, [54] observed that Fe_3_O_4_ NPs caused the highest toxic effects on green alga, as compared to other types of NPs.

Taken together, these data confirm that age likely modulates Fe_3_O_4_ nanoparticles liver toxicity and one might propose that a threshold of toxicity might play a key role. Indeed, as reported by Bakala et al. [35] and possibly explaining our results, the accumulation of deleterious oxidized and carboxymethylated proteins in the matrix concomitant with loss of the IONPs protease activity may affect the ability of aging mitochondria to respond to additional stress. We could therefore speculate that the increase in glycoxidative and oxidative alteration is relevant only if the damage is severe enough to have an impact on mitochondrial function.

However, whether the observed effects are specific of iron oxide in its particulate form or is independent of its size is not known. We therefore submitted old liver mitochondria to iron oxide but not in its particulate form and, interestingly, no deleterious effect was observed. Thus, it is likely that age specifically modulates Fe_3_O_4_ nanoparticles liver toxicity.

## 5. Conclusion

In summary, we demonstrate for the first time that old rats are more susceptible to IONPs nanoparticle exposure in terms of liver mitochondrial respiration and coupling. These age-related changes in liver mitochondrial respiratory chain activity should perhaps be taken into consideration in preclinical and clinical studies of particulate contrast agents.

## Figures and Tables

**Table 1:** Baseline liver mitochondrial respiratory chain complexes activities and mitochondrial coupling.

Control	Young (3 months)	Middle-aged (18 months)
*V*_max⁡_ (µmol O_2_/min/g protein)	29.8 ± 5.6	30.0 ± 1.6
*V*_succ_ (µmol O_2_/min/g protein)	35.0 ± 3.7	33.9 ± 1.7
*V*_tmpd_ (µmol O_2_/min/g protein)	50.0 ± 6.2	43.0 ± 1.6
ACR	9.7 ± 2.6	10.5 ± 1.3

Data are means ± SEM.

ACR: acceptor control ratio (*V*_max⁡_/*V*_0_).

## Abbreviations

NPs: Nanoparticles
IONPs: Iron oxide nanoparticles
*V*_max⁡_: Maximal oxidative capacities
*V*_succ_: Complexes II, III, and IV activities
*V*_tmpd_: Complex IV activity
DEG: Diethylene glycol
ACR: Acceptor control ratio.
